# Metabolic, Familial, and Environmental Factors Associated With Cataract in Young Bangladeshi Adults: A Hospital-Based Matched Case-Control Study

**DOI:** 10.7759/cureus.117208

**Published:** 2026-09-30

**Authors:** Jeenia Afrin, Rehnuma Bushra, Neelufar Sultana Chowdhury, Md. Sadrul Alam Hafiz, Md. Nazrul Islam Chowdhury, Most. Tasnima Akhter, Tanvia Zahin, Isma Johora

**Affiliations:** 1 Department of Biochemistry, Shaheed Suhrawardy Medical College and Hospital, Dhaka, BGD; 2 Department of Biochemistry, Shaheed Tajuddin Ahmad Medical College, Gazipur, BGD; 3 Department of Biochemistry, Dhaka Medical College Hospital, Dhaka, BGD; 4 Department of Pathology, National Institute of Opthalmology and Hospital, Dhaka, BGD; 5 Department of Biochemistry, Sir Salimullah Medical College Mitford Hospital, Dhaka, BGD; 6 Department of Biochemistry, University of Science and Technology Chittagong (USTC), Chattogram, BGD

**Keywords:** bangladesh, cataract, diabetes mellitus, family history, hypertriglyceridemia, hypocalcemia, rural residence, young adults

## Abstract

Background

Cataract is traditionally regarded as a disease of advanced age, but it is also encountered in young adults, in whom the metabolic and biochemical determinants of lens opacification remain poorly characterized. This study aimed to evaluate the sociodemographic, environmental, ocular, metabolic, and biochemical factors associated with cataracts in young Bangladeshi adults.

Methods

In this hospital-based matched case-control study, which was conducted at the National Institute of Ophthalmology and Hospital and the Department of Biochemistry, Dhaka Medical College, between July 2025 and June 2026, 40 adults with ophthalmologist-confirmed cataracts and 40 age- and sex-matched controls without lens opacity, drawn from patients attending the OPD of the same hospital for other eye complaints, were enrolled via purposive sampling. Exposures were recorded through structured face-to-face interviews, and fasting serum calcium (total calcium, not corrected for albumin), 25-hydroxyvitamin D, electrolytes, glucose, and lipid profiles were measured. Independent associations were estimated via multivariable logistic regression with Firth's penalized likelihood to reduce small-sample bias, followed by sensitivity analyses.

Results

Cases and controls had an identical mean age (37.4 ± 8.0 years) and sex distribution (23/40, 57.5% female in each group). Cataract was significantly associated with rural residence (16/40, 40.0% vs 4/40, 10.0%), biomass fuel use (28/40, 70.0% vs 14/40, 35.0%), diabetes mellitus (13/40, 32.5% vs 1/40, 2.5%), and family history of cataracts (21/40, 52.5% vs 5/40, 12.5%; all p < 0.01). Mean serum calcium, sodium, and potassium levels were lower, and fasting glucose levels were higher among cases (all p < 0.05). Hypocalcemia based on total serum calcium (13/40, 32.5% vs 3/40, 7.5%) and hypertriglyceridemia (21/40, 52.5% vs 8/40, 20.0%) were more common among cases, whereas vitamin D status and cholesterol fractions did not differ. After adjustment, hypocalcemia (adjusted OR (aOR) 17.64, 95% CI 3.08-147.60), diabetes mellitus (aOR 15.71, 95% CI 1.93-276.31), family history (aOR 11.76, 95% CI 2.77-69.20), hypertriglyceridemia (aOR 8.51, 95% CI 1.99-52.69), and rural residence (aOR 6.55, 95% CI 1.04-59.96) remained independently associated with cataracts, although CIs were wide, particularly for diabetes mellitus, reflecting the small sample size. The metabolic and familial associations persisted in all sensitivity analyses, including one restricted to urban participants, whereas the association with rural residence was of borderline significance in the main model and did not remain significant in any of the three sensitivity analyses in which it could be assessed; therefore, it should be interpreted cautiously.

Conclusions

Low total serum calcium (hypocalcemia), diabetes mellitus, family history of cataracts, and hypertriglyceridemia were independently associated with early-onset cataracts in young Bangladeshi adults. Without albumin correction or ionized calcium measurement, the hypocalcemia finding is preliminary. The association with rural residence was not robust in sensitivity analyses, possibly reflecting control selection. If confirmed, fasting glucose, serum calcium, and triglyceride levels may help identify young adults for closer eye examination. These case-control findings indicate associations, not causation, and require prospective confirmation.

## Introduction

Cataract is the leading cause of avoidable blindness worldwide, and the Global Burden of Disease Study 2021 confirmed that it still accounts for the largest share of years lived with disability attributable to vision loss across 204 countries and territories [1]. The burden is grossly unequal: South Asia has the highest regional burden of blindness and vision impairment in Asia, with cataracts responsible for approximately one-third of the associated disability-adjusted life-years [2]. In Bangladesh, unoperated cataracts remain the dominant cause of blindness among adults, and cataract surgical coverage remains incomplete [3].

What is less widely appreciated is how young many Bangladeshi cataract patients are. In a hospital-based series from rural Bangladesh, 14% of patients who underwent cataract surgery were aged between 18 and 45 years, whereas only 3.8% of cataract surgical patients in the United States were aged 20 to 49 years [4]. A comparable excess of presenile cataract has been reported elsewhere in South Asia [5]. Because these patients are in the most economically productive phase of life, the consequences of vision loss extend far beyond the individual to their households and national productivity. Surgery, however, addresses the end point rather than the process. It does nothing to protect the fellow eye, the next patient, or the next generation. Prevention demands knowing which exposures actually drive cataractogenesis in young people, and for Bangladesh, such evidence is largely absent.

The mechanistic plausibility of early lens opacification in this population is nonetheless strong. Lens transparency depends on an orderly fiber-cell architecture, stable soluble crystallins, tightly regulated water and ion fluxes, and an intact glutathione-based antioxidant defense; oxidative injury to any of these systems precipitates protein aggregation and light scattering [6]. Several exposures that converge on this pathway are unusually prevalent in Bangladesh. Diabetes is a firmly established cataract risk factor that operates through polyol pathway flux, osmotic stress, and glycation [7,8] and now affects a substantial and increasing proportion of Bangladeshi adults [9]. Dyslipidemia and metabolic syndrome have been linked to cataracts in recent meta-analytic and clinic-based studies [10,11]. Chronic hypocalcemia can precipitate rapidly progressive bilateral cataracts [12,13], possibly because calcium governs lens epithelial ion transport and Na+/K+-ATPase activity, while calcium overload activates calpain-mediated crystallin proteolysis [14,15]. Inherited crystallin and connexin variants explain a meaningful proportion of early-onset disease [16], whereas corticosteroid exposure [17] and uveitis [18] account for additional iatrogenic and inflammatory cases. Environmentally, many Bangladeshi households still cook with biomass, and long-term solid-fuel use is associated with major eye diseases [19,20], while solar ultraviolet radiation remains a recognized contributor [21]. Behavioral and socioeconomic gradients add further complexity [22,23].

These exposures have rarely been examined together in a single young Bangladeshi population, and biochemical determinants, particularly serum calcium, electrolytes, and vitamin D, have been largely neglected. As a result, clinicians in Bangladesh have no local evidence base on which to decide whom to screen, what to measure, or what to counsel when a 35-year-old presents with lens opacity, even as the national metabolic and environmental risk profile deteriorates. We therefore conducted a hospital-based matched case-control study comparing young adults with cataracts with age- and sex-matched controls without cataracts across sociodemographic, environmental, ocular, metabolic, and biochemical domains to identify factors, several of them potentially modifiable, that are independently associated with early-onset cataracts.

## Materials and methods

Study design, setting, and participants

We conducted a hospital-based matched case-control study to examine the metabolic, familial, and environmental correlates of early-onset cataracts among young Bangladeshi adults. Recruitment took place between July 2025 and June 2026 at the National Institute of Ophthalmology and Hospital (NIOH), Dhaka, a national tertiary referral center for eye care, with all biochemical testing carried out in the Clinical Biochemistry Laboratory of the Department of Biochemistry, Dhaka Medical College. This report followed the Strengthening the Reporting of Observational Studies in Epidemiology (STROBE) guidelines for case-control studies. Ethical clearance was obtained from the institutional ethics review committee, and written informed consent was obtained from every participant before enrollment.

Adults aged 18-45 years who presented to the NIOH were screened for eligibility. Cataract status was established by an ophthalmologist through slit-lamp examination, supplemented as needed by fundus examination and visual acuity testing; any lens opacity in either eye satisfied the case definition. Cataract type and grade were not recorded, so cases were not classified by morphology or severity. Forty consecutive patients with confirmed cataracts constituted the case group, and 40 patients free of lens opacity, individually matched to cases by age and sex, formed the comparison group. Controls were recruited from adults aged 18-45 years who attended the NIOH outpatient department during the same period for eye complaints other than cataract, and the absence of lens opacity in both eyes was confirmed by the same ophthalmological examination used for the cases. Each control was of the same sex and the same age in completed years as the corresponding case, and the same exclusion criteria were applied to both groups. Hospital visitors, patient attendants, and community members were not recruited as controls. Both groups were assembled through purposive sampling, with matching applied prospectively as recruitment proceeded. Because participants were selected according to whether or not they had cataracts, the study follows a matched case-control design, and the ORs reported here compare the odds of exposure between cases and controls.

Individuals were excluded for congenital cataract, prior ocular trauma or intraocular surgery (although minor adnexal procedures such as chalazion excision were allowed), a systemic condition known to directly compromise lens clarity (for example, Wilson disease or galactosemia), pregnancy or lactation, calcium or vitamin D supplementation in the preceding six months, chronic kidney or liver disease, or refusal to provide consent.

Sample size

The group sizes were derived from a comparison of two independent proportions using the standard formula for a two-sided test of the difference between two proportions, which is the appropriate basis for a case-control design in which the proportion exposed is compared between cases and controls [24]:

\[ n = \frac{\left(Z_{1-\alpha/2} + Z_{1-\beta}\right)^2 \left[p_1(1-p_1) + p_2(1-p_2)\right]} {\left(p_1-p_2\right)^2} \]

where n is the required sample size per group, p₁ is the anticipated proportion of hypocalcemia among cataract cases (0.35), p₂ is the anticipated proportion among non-cataract controls (0.10), Z₁₋α/₂ = 1.96 corresponds to a two-sided significance level of 0.05, and Z₁₋β = 0.84 corresponds to 80% power. Substituting these values:

\[ n = \frac{(1.96 + 0.84)^2 \left[(0.35 \times 0.65) + (0.10 \times 0.90)\right]} {(0.35 - 0.10)^2} = \frac{7.84 \times 0.3175}{0.0625} = 39.87 \]

This was rounded up to 40 participants per group, giving a total sample of 80 participants. The anticipated proportions were based on previously reported associations between hypocalcemia and cataract formation [12,13].

Data collection

A pretested, semi-structured interview schedule was administered face-to-face to capture sociodemographic details (age, sex, residential locality, monthly household income, level of education), lifestyle and environmental exposures (average daily sunlight exposure, smoking history, use of biomass cooking fuel at home, habitual dietary calcium intake), comorbid conditions (diabetes mellitus, hypertension), corticosteroid exposure, relevant ocular history (uveitis, glaucoma), and any first-degree family history of cataracts. Family history was recorded as cataract in any first-degree relative, irrespective of the age at which the relative developed it.

Laboratory methods

Venous blood (5 mL) was drawn from the antecubital fossa under aseptic conditions after an overnight fast of at least eight hours. The samples were left to clot for 30 minutes and then centrifuged at 3000 rpm for 10 minutes; the separated serum was either processed on the day of collection or stored at -20°C pending analysis. Total serum calcium was assayed by the o-cresolphthalein complexone colorimetric endpoint method on a semiautomated analyzer, 25-hydroxyvitamin D was measured via a competitive ELISA, and sodium and potassium were measured using an ion-selective electrode. Fasting glucose, total cholesterol, triglycerides, and high-density lipoprotein cholesterol were measured enzymatically on an automated analyzer, while low-density lipoprotein cholesterol was calculated using the Friedewald formula. Quality-control samples accompanied every analytical batch.

Definitions and classification of variables

Hypocalcemia was defined as a total serum calcium concentration of less than 8.5 mg/dL [25]. Serum albumin was not measured, so calcium could not be corrected for albumin, and hypocalcemia was therefore based on total rather than albumin-corrected or ionized calcium. Vitamin D status was classified as deficient (below 20 ng/mL), insufficient (20-29.9 ng/mL), or sufficient (30 ng/mL or above), following the Endocrine Society clinical practice guideline [26]. Serum sodium levels of less than 135 or greater than 145 mEq/L were used to define hyponatremia and hypernatremia, respectively, and potassium levels of less than 3.5 or greater than 5.0 mEq/L were used to define hypokalemia and hyperkalemia, respectively [27]. Fasting glucose was categorized as normal (up to 6.0 mmol/L), impaired (6.1-6.9 mmol/L), or in the diabetic range (7.0 mmol/L or above), in accordance with the WHO/International Diabetes Federation criteria [28]. Dyslipidemia was defined according to the National Cholesterol Education Program Adult Treatment Panel III thresholds [29]: total cholesterol at or above 200 mg/dL, triglycerides at or above 150 mg/dL, low-density lipoprotein (LDL) cholesterol at or above 130 mg/dL, and high-density lipoprotein (HDL) cholesterol under 40 mg/dL in men or under 50 mg/dL in women. Corticosteroid use was defined as systemic, inhaled, or topical ocular therapy sustained for more than two consecutive weeks within the prior six months. Daily sunlight exposure, averaged over the preceding three months for the 10:00 to 16:00 window, was classified as low (under 1 hour), moderate (1 to 3 hours), or high (over 3 hours). Smoking status was recorded as current, former, or never. Education and household income followed the Bangladesh Bureau of Statistics categories [30], and habitual dietary calcium intake was rated by the interviewer as inadequate, moderate, or adequate on the basis of reported consumption of milk, dairy products, small bony fish, and green leafy vegetables. Corticosteroid exposure, daily sunlight exposure, and habitual dietary calcium intake were operational definitions specific to this study, as no standard classification exists for these exposures.

Statistical analysis

Data were analyzed using SPSS version 26 (IBM Corp., Armonk, NY, USA) and Stata version 17 (StataCorp LLC, College Station, TX, USA). Continuous variables were presented as mean ± SD and compared using the independent-samples t-test with Welch's correction or the Mann-Whitney U test for skewed variables. Categorical variables were presented as frequencies and percentages and compared using Pearson's chi-square test or Fisher's exact test when expected counts were less than five. Bivariable p values were additionally adjusted using the Benjamini-Hochberg false discovery rate procedure and the Holm correction. Six exposures, chosen on the basis of prior literature and the bivariable results (rural residence, biomass fuel use, diabetes mellitus, family history of cataract, hypocalcemia, and hypertriglyceridemia), were entered into a multivariable logistic regression model fitted using Firth's penalized likelihood. aORs with penalized profile-likelihood 95% CIs were reported. Because matching was limited to age and sex, unconditional logistic regression was applied. Model calibration was assessed using the Hosmer-Lemeshow test. Sensitivity analyses were performed by restricting the analysis to urban participants, excluding one extreme triglyceride value (957 mg/dL), restricting the analysis to participants without diabetes mellitus, and additionally adjusting for age and sex. A p value < 0.05 was considered statistically significant.

Ethics

The protocol was approved by the Institutional Review Board of Dhaka Medical College, Dhaka (approval no: IRB-DMC/2025/154), and institutional permission was obtained from the NIOH. Written informed consent was obtained from every participant in Bangla after a full explanation of the purpose, procedures, risks, and benefits of the study. Participation was voluntary, and participants could withdraw at any time. The data were anonymized and stored securely.

## Results

Sociodemographic, lifestyle, and environmental characteristics are shown in Table 1. Eighty participants were enrolled, comprising 40 with cataracts and 40 without cataracts. Because matching was performed at the individual level, the two groups were identical in terms of age (mean 37.4 ± 8.0 years; range 19-45 years) and sex (23 women and 17 men in each group; 57.5% female). Because age and sex were matching factors, their distributions cannot be interpreted as risk factors, and the female predominance among cases reflects the composition of the case series rather than a sex difference in risk. The median age of the cases was 40 years (IQR 35-44), and 33 of the 40 cases (82.5%) were aged 35 years or older. A significantly greater proportion of cataract patients lived in rural areas (16/40, 40.0%) than did non-cataract individuals (4/40, 10.0%; χ² = 9.60, df = 1, p = 0.002). Because controls were recruited from the OPD of a tertiary hospital in Dhaka, this difference may partly reflect the catchment of the control group (see Discussion). Household biomass fuel use was also more common among cataract patients (28/40, 70.0% vs 14/40, 35.0%; χ² = 9.83, df = 1, p = 0.002). Educational attainment showed a gradient in the expected direction, with graduate-level education in 2/40 (5.0%) of cataract patients versus 10/40 (25.0%) of non-cataract individuals, but the overall difference did not reach statistical significance (p = 0.087), as was also the case for family income (p = 0.091) and habitual dietary calcium intake (p = 0.072). Daily sunlight exposure and smoking status did not differ between the groups.

**Table 1:** Sociodemographic, lifestyle, and environmental characteristics of the study participants (N = 80). (a) Pearson’s chi-square test. Age is not tabulated because the groups were individually matched and their age distributions were identical (mean 37.4 ± 8.0 years; range 19-45 years in both groups); age is reported in the text. Expected cell counts of less than five occurred in the comparisons of educational attainment, family income, and smoking status; these p-values should be interpreted with caution. Bold indicates p < 0.05. The test statistic column presents the Pearson chi-square statistic and corresponding degrees of freedom for each comparison. BDT: Bangladeshi taka.

Variable	Cataract (n = 40), n (%)	Non-cataract (n = 40), n (%)	Test statistic	p-value
Sex				
Female	23 (57.5)	23 (57.5)	χ² = 0.000 (df = 1)	1.000 (a)
Male	17 (42.5)	17 (42.5)		
Area of residence				
Urban	24 (60.0)	36 (90.0)	χ² = 9.600 (df = 1)	0.002 (a)
Rural	16 (40.0)	4 (10.0)		
Educational attainment				
Illiterate	5 (12.5)	4 (10.0)	χ² = 8.126 (df = 4)	0.087 (a)
Primary	13 (32.5)	6 (15.0)		
Secondary	12 (30.0)	11 (27.5)		
Higher secondary	8 (20.0)	9 (22.5)		
Graduate and above	2 (5.0)	10 (25.0)		
Monthly family income (BDT)				
<10,000	3 (7.5)	0 (0.0)	χ² = 4.800 (df = 2)	0.091 (a)
10,000-25,000	29 (72.5)	26 (65.0)		
>25,000	8 (20.0)	14 (35.0)		
Daily sunlight exposure				
<1 h	21 (52.5)	17 (42.5)	χ² = 0.990 (df = 2)	0.609 (a)
1-3 h	12 (30.0)	13 (32.5)		
>3 h	7 (17.5)	10 (25.0)		
Smoking status				
Current smoker	10 (25.0)	7 (17.5)	χ² = 1.214 (df = 2)	0.545 (a)
Former smoker	2 (5.0)	4 (10.0)		
Never smoker	28 (70.0)	29 (72.5)		
Household biomass fuel use				
Yes	28 (70.0)	14 (35.0)	χ² = 9.825 (df = 1)	0.002 (a)
No	12 (30.0)	26 (65.0)		
Habitual dietary calcium intake				
Inadequate	26 (65.0)	16 (40.0)	χ² = 5.267 (df = 2)	0.072 (a)
Moderate	11 (27.5)	17 (42.5)		
Adequate	3 (7.5)	7 (17.5)		

Table 2 summarizes comorbidities, ocular history, corticosteroid exposure, and family history. Diabetes mellitus was reported by 13 cataract patients (13/40, 32.5%) and one non-cataract individual (1/40, 2.5%; χ² = 12.47, df = 1, p < 0.001). A family history of cataracts was present in 21 cataract patients (21/40, 52.5%) compared with five non-cataract individuals (5/40, 12.5%; χ² = 14.59, df = 1, p < 0.001). Hypertension, corticosteroid use, uveitis, and glaucoma were more common among cataract patients, but the differences were not statistically significant.

**Table 2:** Comorbidities, ocular history, corticosteroid exposure, and family history (N = 80). (a) Pearson’s chi-square test; (b) Fisher’s exact test. A p value < 0.05 was considered statistically significant. Chi-square statistics with corresponding degrees of freedom are reported for comparisons evaluated using Pearson’s chi-square test. Fisher’s exact test yields an exact p value without a corresponding chi-square test statistic; therefore, these rows are marked as NA in the test statistic column.

Variable	Cataract (n = 40), n (%)	Non-cataract (n = 40), n (%)	Test statistic	p value
Diabetes mellitus	13 (32.5)	1 (2.5)	χ² = 12.468 (df = 1)	<0.001 (a)
Hypertension	4 (10.0)	3 (7.5)	NA	1.000 (b)
Corticosteroid use	4 (10.0)	1 (2.5)	NA	0.359 (b)
Uveitis	3 (7.5)	1 (2.5)	NA	0.615 (b)
Glaucoma	4 (10.0)	2 (5.0)	NA	0.675 (b)
Family history of cataract	21 (52.5)	5 (12.5)	χ² = 14.587 (df = 1)	<0.001 (a)

Continuous serum biochemical measurements are shown in Table 3. The mean serum calcium level was significantly lower in cataract patients than in non-cataract individuals (8.76 ± 0.49 vs 9.00 ± 0.42 mg/dL; mean difference -0.24 mg/dL, 95% CI -0.44 to -0.04; p = 0.022). The mean serum sodium level (139.37 ± 1.80 vs 140.51 ± 2.13 mEq/L; p = 0.011) and mean serum potassium level (4.42 ± 0.32 vs 4.61 ± 0.23 mEq/L; p = 0.003) were also lower among cataract patients, although the means in both groups lay within the reference interval, and the serum calcium level correlated modestly with the serum potassium level across the whole sample (r = 0.33, p = 0.003). The mean serum 25-hydroxyvitamin D concentration did not differ between the groups (23.54 ± 10.53 vs 24.21 ± 8.86 ng/mL; p = 0.760). The mean fasting glucose level was significantly greater in cataract patients (7.11 ± 3.46 vs 5.57 ± 0.25 mmol/L; p = 0.008), a difference confirmed by nonparametric testing (Mann-Whitney p = 0.001). Among the lipid fractions, only triglycerides differed: the median was greater in cataract patients (158.0, IQR 116.0-197.3 mg/dL) than in non-cataract individuals (128.0, IQR 110.5-139.3 mg/dL; Mann-Whitney p = 0.019), whereas the difference in means was not significant (184.60 ± 145.71 vs 135.90 ± 50.00 mg/dL; p = 0.051). One cataract patient had an extreme triglyceride value of 957 mg/dL. In a post hoc sensitivity analysis that excluded this observation, the difference in means was of borderline significance (164.8 ± 75.4 vs 135.9 ± 50.0 mg/dL; p = 0.049). The mean total cholesterol, LDL cholesterol, and HDL cholesterol levels did not differ between the groups.

**Table 3:** Continuous serum biochemical measurements by cataract status (N = 80). Values are presented as mean ± SD unless otherwise stated. (a) Independent-samples t-test with Welch’s correction; (b) Mann-Whitney U test, reported for the two markedly skewed variables; (c) LDL cholesterol values were available for 38 cataract patients and 40 non-cataract individuals. A p-value < 0.05 was considered statistically significant. The test statistic column presents Welch’s t statistic with the corresponding degrees of freedom for variables compared using the t-test and the standardized Z statistic for the two variables compared using the Mann-Whitney U test. HDL: High-density lipoprotein; LDL: Low-density lipoprotein; 25(OH)D: 25-hydroxyvitamin D.

Variable	Cataract (n = 40)	Non-cataract (n = 40)	Mean difference (95% CI)	Test statistic	p value
Serum calcium, mg/dL	8.76 ± 0.49	9.00 ± 0.42	-0.24 (-0.44 to -0.04)	t = -2.352 (df = 76.2)	0.022 (a)
Serum 25(OH)D, ng/mL	23.54 ± 10.53	24.21 ± 8.86	-0.67 (-5.00 to 3.67)	t = -0.308 (df = 75.8)	0.760 (a)
Serum sodium, mEq/L	139.37 ± 1.80	140.51 ± 2.13	-1.14 (-2.02 to -0.27)	t = -2.585 (df = 75.9)	0.011 (a)
Serum potassium, mEq/L	4.42 ± 0.32	4.61 ± 0.23	-0.20 (-0.32 to -0.07)	t = -3.049 (df = 70.8)	0.003 (a)
Fasting glucose, mmol/L	7.11 ± 3.46	5.57 ± 0.25	1.53 (0.42 to 2.65)	t = 2.808 (df = 39.4)	0.008 (a)
Median (IQR)	6.05 (5.40-6.90)	5.60 (5.60-5.70)	-	Z = 3.291	0.001 (b)
Total cholesterol, mg/dL	184.75 ± 41.82	185.75 ± 32.22	-1.00 (-17.64 to 15.64)	t = -0.120 (df = 73.2)	0.905 (a)
Triglycerides, mg/dL	184.60 ± 145.71	135.90 ± 50.00	48.70 (-0.27 to 97.67)	t = 1.999 (df = 48.1)	0.051 (a)
Median (IQR)	158.0 (116.0-197.3)	128.0 (110.5-139.3)	-	Z = 2.346	0.019 (b)
LDL cholesterol, mg/dL (c)	110.77 ± 38.95	115.63 ± 25.34	-4.86 (-19.81 to 10.09)	t = -0.650 (df = 63.1)	0.518 (a)
HDL cholesterol, mg/dL	41.43 ± 5.36	42.97 ± 2.78	-1.54 (-3.45 to 0.37)	t = -1.613 (df = 58.6)	0.112 (a)

The corresponding categorical classifications are shown in Table 4. Hypocalcemia, defined on the basis of total serum calcium without albumin correction, was more than four times more common among cataract patients (13/40, 32.5%) than among non-cataract individuals (3/40, 7.5%; χ² = 7.81, df = 1, p = 0.005). Categorical hyponatremia occurred in a single cataract patient (1/40, 2.5% vs 0/40, 0.0%) and was not significant (p = 1.000), while no participant in either group met the definition of hypokalemia, so no comparison was possible. Vitamin D deficiency was numerically more common among cataract patients (18/40, 45.0%) than among non-cataract individuals (12/40, 30.0%), but the distribution across the three vitamin D categories did not differ significantly (p = 0.323). Glycemic status differed markedly between the groups: all 10 participants with impaired fasting glucose (10/40, 25.0%) and all 10 with fasting glucose in the diabetic range (10/40, 25.0%) belonged to the cataract group, whereas every non-cataract individual had a normal fasting glucose level (40/40, 100.0%; χ² = 26.67, df = 2, p < 0.001). Hypertriglyceridemia was present in 21 cataract patients (21/40, 52.5%) compared with eight non-cataract individuals (8/40, 20.0%; χ² = 9.14, df = 1, p = 0.003) and was the only lipid abnormality that differed significantly; high total cholesterol (14/40, 35.0% vs 8/40, 20.0%; p = 0.133), high LDL cholesterol (9/38, 23.7% vs 6/40, 15.0%; p = 0.331), and low HDL cholesterol (31/40, 77.5% vs 26/40, 65.0%; p = 0.217) were numerically, but not significantly, more common among cataract patients.

**Table 4:** Categorical biochemical classifications by cataract status (N = 80). (a) Pearson’s chi-square test; (b) Fisher’s exact test; (c) denominator of 38 for the cataract group because two LDL values were missing; (d) <40 mg/dL in men and <50 mg/dL in women. The p values shown for vitamin D status and glycemic status are from the three-category comparisons; comparison of vitamin D deficiency with non-deficiency (insufficient plus sufficient) yielded p = 0.166. Hypokalemia was not present in any participant and was therefore not tabulated. A p value < 0.05 was considered statistically significant. The test statistic column gives the Pearson chi-square value with its degrees of freedom; rows tested by Fisher’s exact test yield no test statistic and are marked NA. HDL: High-density lipoprotein; LDL: Low-density lipoprotein; NA: Not applicable; df: Degrees of freedom.

Classification	Cataract (n = 40), n (%)	Non-cataract (n = 40), n (%)	Test statistic	p value
Hypocalcemia (<8.5 mg/dL)	13 (32.5)	3 (7.5)	χ² = 7.812 (df = 1)	0.005 (a)
Vitamin D status				
Deficient (<20 ng/mL)	18 (45.0)	12 (30.0)	χ² = 2.259 (df = 2)	0.323 (a)
Insufficient (20-29.9 ng/mL)	14 (35.0)	20 (50.0)		
Sufficient (≥30 ng/mL)	8 (20.0)	8 (20.0)		
Hyponatremia (<135 mEq/L)	1 (2.5)	0 (0.0)	NA	1.000 (b)
Glycemic status				
Normal (≤6.0 mmol/L)	20 (50.0)	40 (100.0)	χ² = 26.667 (df = 2)	<0.001 (a)
Impaired fasting glucose (6.1-6.9 mmol/L)	10 (25.0)	0 (0.0)		
Diabetic range (≥7.0 mmol/L)	10 (25.0)	0 (0.0)		
High total cholesterol (≥200 mg/dL)	14 (35.0)	8 (20.0)	χ² = 2.257 (df = 1)	0.133 (a)
High triglycerides (≥150 mg/dL)	21 (52.5)	8 (20.0)	χ² = 9.141 (df = 1)	0.003 (a)
High LDL cholesterol (≥130 mg/dL) (c)	9 (23.7)	6 (15.0)	χ² = 0.946 (df = 1)	0.331 (a)
Low HDL cholesterol (d)	31 (77.5)	26 (65.0)	χ² = 1.526 (df = 1)	0.217 (a)

Six variables were entered into the penalized logistic regression model (Table 5). Five factors were independently associated with cataracts: hypocalcemia (aOR 17.64, 95% CI 3.08-147.60; p < 0.001), diabetes mellitus (aOR 15.71, 95% CI 1.93-276.31; p = 0.007), family history of cataracts (aOR 11.76, 95% CI 2.77-69.20; p < 0.001), hypertriglyceridemia (aOR 8.51, 95% CI 1.99-52.69; p = 0.003), and rural residence (aOR 6.55, 95% CI 1.04-59.96; p = 0.046), the last of which was of borderline significance. Biomass fuel use, which was strongly associated with cataracts in the bivariable analysis (crude OR 4.33, 95% CI 1.70-11.07), lost its association after adjustment (aOR 1.41, 95% CI 0.33-5.97; p = 0.637). Penalization shrank each of these adjusted estimates relative to a conventional maximum-likelihood fit of the same model but left the direction of every association, the ordering of the variables, and the overall conclusions unchanged. The penalized model was globally significant (penalized likelihood-ratio chi-square 56.26, df = 6, p < 0.001) and calibrated adequately (Hosmer-Lemeshow chi-square 7.32, df = 8, p = 0.502, using ten equal-sized groups of predicted probability). The CIs nevertheless remained wide, particularly for diabetes mellitus, because only one non-cataract individual was diabetic; the lower confidence limits, rather than the point estimates, are a more reliable guide to the minimum magnitude of each association. The estimate for diabetes mellitus, in particular, was unstable, with an upper confidence limit above 270 even after penalization, and its point estimate should not be interpreted as a precise measure of effect size.

**Table 5:** Penalized multivariable logistic regression of factors associated with cataracts (N = 80). Dependent variable: cataract (1 = present, 0 = absent). The reference category for each factor is the absent or normal condition; rows are ordered by the magnitude of the penalized adjusted odds ratio. Penalized model: likelihood-ratio chi-square = 56.26, df = 6, p < 0.001; Hosmer-Lemeshow chi-square = 7.32, df = 8, p = 0.502 (ten equal-sized groups of predicted probability). A p value < 0.05 was considered statistically significant. The test statistic column gives the penalized likelihood-ratio chi-square statistic with 1 degree of freedom for each factor. † For factors with p < 0.001, the tabulated value represents the lower bound corresponding to p = 0.001. aOR: adjusted odds ratio.

Factor	Crude OR (95% CI)	Firth-penalized aOR (95% CI)	Test statistic	p-value
Hypocalcemia (vs normocalcemia)	5.94 (1.54-22.90)	17.64 (3.08-147.60)	χ² = 11.18 (df = 1)	<0.001
Diabetes mellitus (vs none)	18.78 (2.32-152.17)	15.71 (1.93-276.31)	χ² = 7.20 (df = 1)	0.007
Family history of cataract (vs none)	7.74 (2.51-23.81)	11.76 (2.77-69.20)	χ² = 11.93 (df = 1)	<0.001
Hypertriglyceridemia (vs normal)	4.42 (1.64-11.93)	8.51 (1.99-52.69)	χ² = 8.84 (df = 1)	0.003
Rural residence (vs urban)	6.00 (1.79-20.15)	6.55 (1.04-59.96)	χ² = 4.00 (df = 1)	0.046
Household biomass fuel use (vs none)	4.33 (1.70-11.07)	1.41 (0.33-5.97)	χ² = 0.22 (df = 1)	0.637

The sensitivity analyses are shown in Table 6. When the model was restricted to the 60 urban participants (24 cases and 36 controls), hypocalcemia (aOR 12.81, 95% CI 2.19-105.40), diabetes mellitus (aOR 10.89, 95% CI 1.30-193.20), family history of cataracts (aOR 7.51, 95% CI 1.54-48.29), and hypertriglyceridemia (aOR 6.80, 95% CI 1.69-38.10) remained associated with cataracts, and biomass fuel use remained unassociated (aOR 1.52, 95% CI 0.36-6.17). Hypocalcemia, family history of cataracts, and hypertriglyceridemia also remained associated with cataracts after exclusion of the participant with the extreme triglyceride value, among the 66 participants without diabetes mellitus, and after additional adjustment for age and sex; diabetes mellitus remained associated in every model in which it was included. In contrast, rural residence was not significantly associated with cataracts after exclusion of the extreme triglyceride value (aOR 6.14, 95% CI 0.96-56.79; p = 0.056), among participants without diabetes mellitus (aOR 5.93, 95% CI 0.87-57.32; p = 0.071), or after adjustment for age and sex (aOR 3.88, 95% CI 0.52-39.79; p = 0.193).

**Table 6:** Sensitivity analyses of the penalized multivariable logistic regression model (N = 80). Values are Firth-penalized adjusted odds ratios with penalized profile-likelihood 95% CIs, and p values are from penalized likelihood-ratio tests. Each model contains the factors shown in Table 5, except that rural residence is omitted from the model restricted to urban participants and diabetes mellitus is omitted from the model restricted to participants without diabetes mellitus because these factors do not vary within those subgroups (shown as not applicable). The extreme triglyceride value was 957 mg/dL. In the model additionally adjusted for the matching factors, the aOR per 10-year increase in age was 0.46 (95% CI 0.17-1.13), and the aOR for male sex was 0.59 (95% CI 0.15-2.14). A p value < 0.05 was considered statistically significant. aOR: adjusted odds ratio.

Factor	Urban participants only (n = 60)	Excluding the extreme triglyceride value (n = 79)	Participants without diabetes mellitus (n = 66)	Additionally adjusted for age and sex (N = 80)
Hypocalcemia (vs normocalcemia)	12.81 (2.19-105.40), p = 0.004	19.14 (3.32-162.08), p < 0.001	16.47 (2.79-140.33), p = 0.002	21.44 (3.51-192.46), p < 0.001
Diabetes mellitus (vs none)	10.89 (1.30-193.20), p = 0.026	15.67 (2.00-266.03), p = 0.006	Not applicable	16.69 (2.01-354.11), p = 0.006
Family history of cataract (vs none)	7.51 (1.54-48.29), p = 0.012	12.66 (2.97-74.36), p < 0.001	11.21 (2.65-65.58), p < 0.001	9.87 (2.24-60.18), p = 0.002
Hypertriglyceridemia (vs normal)	6.80 (1.69-38.10), p = 0.006	7.00 (1.59-43.74), p = 0.009	7.78 (1.73-50.24), p = 0.006	13.64 (2.76-99.82), p < 0.001
Rural residence (vs urban)	NA	6.14 (0.96-56.79), p = 0.056	5.93 (0.87-57.32), p = 0.071	3.88 (0.52-39.79), p = 0.193
Household biomass fuel use (vs none)	1.52 (0.36-6.17), p = 0.555	1.68 (0.38-7.49), p = 0.488	1.45 (0.33-6.21), p = 0.617	2.12 (0.43-10.82), p = 0.350

## Discussion

In this hospital-based matched case-control study of 40 young Bangladeshi adults with cataracts and 40 exactly age- and sex-matched non-cataract individuals, five factors, namely, hypocalcemia (defined on the basis of total serum calcium), diabetes mellitus, a positive family history of cataracts, hypertriglyceridemia, and, less robustly, rural residence, were independently associated with lens opacity. Together, these results produced a regression model that was globally significant and adequately calibrated, although the confidence intervals around each estimate remained wide throughout the analysis. These findings suggest that early-onset cataracts in this population are associated with a small number of identifiable metabolic, hereditary, and environmental exposures, although the study design does not allow any of them to be regarded as a cause.

Diabetes mellitus showed the second strongest adjusted association (aOR 15.71), although this was also the least precise estimate in the model (95% CI 1.93-276.31) because only one control participant was diabetic. Every participant with impaired fasting glucose or diabetic-range fasting glucose was a case, and the mean fasting glucose level was 1.5 mmol/L higher among the cases. This magnitude exceeds that reported in a recent meta-analysis of 30 studies [7], which may partly reflect the smaller competing contribution of aging to lens opacification in young adults, although the wide confidence interval means that the true magnitude could be considerably smaller. Sustained hyperglycemia drives aldose reductase-mediated sorbitol accumulation, osmotic swelling of fiber cells, and secondary oxidative injury [8]. With diabetes and prediabetes now affecting roughly one-fifth of Bangladeshi adults and increasing [9], this association deserves confirmation in prospective studies, but our data cannot show that diabetes causes presenile cataracts or estimate what share of such cataracts it accounts for nationally.

Hypertriglyceridemia was the only lipid abnormality independently associated with cataracts (aOR 8.51), whereas total, LDL, and HDL cholesterol were not associated with cataracts according to either continuous or categorical analyses. This selective pattern mirrors recent evidence linking cataracts more consistently to metabolic syndrome and triglyceride-predominant dyslipidemia than to hypercholesterolemia [10,11] and is biologically coherent, since triglyceride excess tracks insulin resistance and promotes lipid peroxidation of lens membranes [6]. This association persisted after exclusion of the participant with an extreme triglyceride value (aOR 7.00) and among participants without diabetes mellitus (aOR 7.78). Nevertheless, the difference in mean triglyceride concentration was not significant in the full sample, and hypertriglyceridemia may partly mark underlying insulin resistance, which was not measured, rather than act directly on the lens. This finding should therefore be regarded as hypothesis-generating.

Hypocalcemia showed the strongest adjusted association (aOR 17.64), and the mean serum calcium level was 0.24 mg/dL lower among the patients. However, this estimate was imprecise (95% CI 3.08-147.60) and rests on total serum calcium alone. Case reports of rapidly progressive bilateral cataracts in patients with hypoparathyroidism establish that severe hypocalcemia is cataractogenic [12,13]; our findings are compatible with an association that extends to mild, subclinical reductions in a young hospital-based population. This interpretation requires caution because serum albumin was not measured, and some participants classified as hypocalcemic on the basis of total calcium may have had normal ionized calcium, so misclassification in either direction cannot be excluded. In addition, the difference in mean serum calcium did not remain significant after correction for multiple comparisons. Calcium regulates lens epithelial ion transport and Na+/K+-ATPase activity, and disturbance of this system permits sodium and water influx, fiber-cell swelling, and calpain-mediated crystallin proteolysis [14]. Consistent with a generalized ionic disturbance rather than isolated calcium deficiency, the mean serum sodium and potassium levels were also modestly lower in the cases than in the controls, and the serum calcium level was correlated with potassium. Because these means remained within the reference interval and categorical hyponatremia and hypokalemia were virtually absent, we interpret the electrolyte findings as supportive rather than causal, in keeping with the coupled ionic homeostasis that maintains lens transparency [15]. Taken together, our data indicate an association between cataract and low total serum calcium rather than proven hypocalcemia, and this finding should be confirmed with albumin-corrected or ionized calcium measurements before any clinical inference is drawn.

A family history of cataracts was associated with approximately twelvefold adjusted odds, which is consistent with the recognized contribution of crystallin and connexin variants to early-onset cataracts [16] and may point to a genetically susceptible subgroup that presented decades before age-related disease. This interpretation remains tentative, however, because family history was recorded without distinguishing presenile from age-related cataract in relatives, and common senile cataract in older family members may account for part of the positive histories.

The environmental findings require careful interpretation. Biomass fuel use was strongly associated with cataracts in the crude analysis (OR 4.33) but lost its association after adjustment (aOR 1.41), whereas rural residence retained an adjusted association of borderline significance (aOR 6.55, 95% CI 1.04 to 59.96; p = 0.046). Because biomass cooking is nearly universal in rural Bangladeshi households, residence and fuel use are collinear, and residence may capture wider rural exposure profiles, including outdoor labor, nutritional inadequacy, and delayed presentation, of which solid-fuel smoke is one component [19,20]. This finding does not contradict cohort evidence linking solid fuel to eye disease [19]; it indicates that our design cannot separate the two. The rural disadvantage in cataract detection and treatment is well described in South Asia [23]. A further explanation lies in the source of controls. Because controls were outpatients at a tertiary eye hospital in Dhaka, they may have been more likely than cases to live in the city, and part of the rural excess among cases may therefore reflect referral patterns rather than an effect of residence itself. Consistent with this, rural residence was the least robust association in the sensitivity analyses, whereas hypocalcemia, diabetes mellitus, family history, and hypertriglyceridemia remained associated with cataracts when the analysis was restricted to urban participants. Rural residence, by contrast, did not remain significantly associated with cataracts after exclusion of the extreme triglyceride value, among participants without diabetes mellitus, or after adjustment for age and sex. We therefore regard this association as tentative and do not interpret it as evidence that rural residence itself increases the risk of early-onset cataract.

Sunlight exposure, smoking, education, income, hypertension, steroid use, uveitis, glaucoma, and vitamin D status showed no significant associations, most plausibly reflecting the limited statistical power for relatively uncommon exposures rather than a true absence of any effect [21,22].

The present study has some strengths. To the best of our knowledge, it is one of the first studies in Bangladesh to examine metabolic, biochemical, familial, and environmental factors together in young adults with cataract, and it used penalized regression with several sensitivity analyses. However, our study had some limitations. First, as a case-control study, it cannot establish causality, and the ORs do not represent prevalence or risk. Second, the small sample produced imprecise estimates, particularly for diabetes mellitus, and the six model exposures coincided with those significant in bivariable analysis, so the adjusted estimates may be optimistic. Third, purposive recruitment from a single hospital, with outpatient controls, may have introduced selection bias and exaggerated the association with rural residence, which was also collinear with biomass fuel use. Fourth, several exposures were self-reported, and family history did not distinguish presenile from age-related cataract. Fifth, cataract type and grade were not recorded. Sixth, the mean triglyceride difference was significant only after post hoc exclusion of one extreme value, and the mean serum calcium difference did not survive correction for multiple testing. Finally, albumin, ionized calcium, parathyroid hormone, magnesium, HbA1c, and oxidative stress markers were not measured, so hypocalcemia was classified on the basis of total serum calcium alone and may have been misclassified in either direction, which limits the interpretation of the strongest association in the model. Vitamin D was also measured by ELISA without recorded kit details. Larger prospective population-based studies are needed to confirm these findings.

## Conclusions

The present study showed that low total serum calcium (hypocalcemia), diabetes mellitus, a family history of cataract, and hypertriglyceridemia were independently associated with increased odds of early-onset cataract among young Bangladeshi adults. Because albumin-corrected and ionized calcium were not measured, the association with hypocalcemia should be regarded as preliminary and requires confirmation. Rural residence was also associated with increased odds in the main model, but this association was of borderline significance, was not robust in sensitivity analyses, and may partly reflect how the controls were selected, whereas household biomass fuel use was not independently associated after adjustment. Lower serum calcium, sodium, and potassium levels and higher fasting glucose levels were associated with cataract in bivariable analyses. Because of the case-control design and small sample size, these findings reflect associations rather than causation. Further studies, especially population-based cohort research with albumin-corrected or ionized calcium measurements, are needed to provide more conclusive evidence regarding these associations.
